# Effect of feeding mulberry leaves on nutrient digestibility, carcass characteristics, blood parameters and productive performance of growing Flanders rabbits

**DOI:** 10.1007/s11250-025-04727-7

**Published:** 2026-01-20

**Authors:** Hanan A. M. Hassanien, Nabila M. Elkassas, Rehab A. Mohamed, Ezat El-Bltagy, Sherine Fayad, Soad El Naggar, Abdelfattah Z. M. Salem

**Affiliations:** 1 Animal Production Research Institute, Agricultural Research Center, Giza, Egypt; 2 Desert Research Center, Genetic Resources Department, Cairo, Egypt; 3 Animal Production Department, National Research Centre, Dokki, Giza, 12622 Egypt; 4 Faculty of Veterinary Medicine and Zootechnics, Autonomous University of the State of Mexico, Toluca, 50000 Mexico; 5 Dipartimento di Scienze del Suolo, della Pianta e degli Alimenti (Di.S.S.P.A.), Università degli Studi di Bari, Via Giovanni Amendola, 165/a, 70126 Bari, BA Italy

**Keywords:** Morus Alba, Leaves, Rabbits, Growth performance, Blood constituents, Economic efficiency

## Abstract

This study was conducted to evaluate the effect of mulberry leaves (ML) as an alternative source of clover hay on growth performance, nutrient digestibility, carcass traits, and blood parameters of Flanders rabbits. Thirty-six growing rabbits were randomly and equally divided to be fed three diets as follows: basal diet (control, ML0), basal diet with mulberry leaves as a replacement for clover hay by either 9% (ML9) or 12% (ML12) for 63 days as growth trials. Three animals in each group were slaughtered to assess the carcass traits and determine some blood parameters at the end of the experiment. Results indicated that ML decreased (*P* < 0.05) dry and organic matter digestibility and increased ether extract digestibility compared to the control group. At the same time, there were no significant differences in the other nutrients’ digestibility and nutritive value, such as digestible crude protein, among groups. The discrepancies between feeding groups in final body weight, average daily gain, and dressing percentage lacked significant. All blood parameters were in the normal range, where the cholesterol, triglyceride, total lipids, and antioxidant capacity were recorded at the lower concentration (*P* < 0.05) for ML9 and ML12 compared with ML0. Additionally, using mulberry leaves at a 12% substitution decreased the feed cost and increased the economic efficiency. It can be concluded that mulberry leaves can be used in place of clover hay in growing rabbits’ diets up to 12%.

## Introduction

In developing nations, industrialization and population growth have increased the lack of land for agriculture and foraging, causing the search of inexpensive, rich in nutrients feed (Klinger 2018). Clover hay is optimal for rabbit nutrition; nevertheless, its high cost and low yield limit its economic advantages, necessitating the inclusion of alternative roughages. Mulberry (*Morus* spp.) is a rapidly growing deciduous tree. It can flourish in both temperate and tropical regions and is utilized in the food, feed, and silk sectors. Mulberry leaves contain a significant protein content (15–35%) and are rich in essential amino acids such as leucine (1.45–2.58%) and lysine (1.11–1.80%) - (Al-Kirshi et al. 2009; Wang et al. 2017). They are very palatable and easily digestible (Yupakarn et al. 2015), and numerous research have examined them as an alternative protein source for animal feed (Muthoni et al. 2020; Ustundag and Ozdogan 2023). The ML contains bioactive compounds such as saponins, tannins, carotenoids, and polyphenols, which enhance immune function, combat bacteria, and reduce blood sugar levels (Chen et al. 2019; Dhiman et al. 2020). Excessive use of these substances may introduce anti-nutritional factors such as tannins and elevated crude fiber, which can impede nutrient absorption in the body (Cai et al. 2019; Ding et al. 2021). Hou et al. (2020) shown that the inclusion of ML below 15% improved rabbit growth and meat quality, whereas 20% reduced performance. Premalatha et al. (2012) also found that mulberry leaves resembled lucerne leaves in rabbit diets. The FAO states that mulberry leaves may serve as an alternative to cereals for cow feed. This study seeks to evaluate the effects of replacing clover hay with mulberry leaves on the growth, nutrient digestibility, carcass traits, and blood parameters of growing rabbits.

## Materials and methods

The El-Gemmaiza Animal Production Research Station, a facility owned by the Animal Production Research Institute (APRI), Agriculture Research Center (ARC), and the Egyptian Ministry of Agriculture’s, El-Gharbia Governorate, was the site of this study. All in vivo trials involving animals were approved by the Institutional Animal Care and Use Committee (ARC-IACUC), Agriculture Research Centre, Egypt (Protocol Number ARC-AP- 22–27). In this experiment, the rabbits slaughtered were handled humanely.

### Preparation of mulberry powder

A moisture content of less than 8% was attained by drying fresh mulberry leaves (ML) at 60 °C for 4 days. After the leaves had dried, they were processed into mulberry leaf powder using a grinder that included a sieve with holes of 1.5 mm in diameter.

### Experimental animals, management and feeding

Weaning Flanders rabbits were raised in El-Gemmaiza Experimental Research Station. After being weaned at 6 weeks of age, thirty of them were equally divided into three groups, based on their average initial live body weight (607*+* 46 g). Mulberry leaf powder was substituted with clover hay at 3 levels: 0, 9, and 12%, representing the control (ML0), ML9, and ML12 groups, respectively. The experimental diets were presented in the form of pellets and designed to be roughly isocaloric, isonitrogenous, and iso-fibrous. Table 1 lists the ingredients of the three experimental pelleted diets. Mulberry leaves, clover hay and experimental diets as dry matter, crude protein, crude fibre, ether extract and ash were analyzed according to the Association of Official Analytical Chemists (AOAC 2000) procedures, while nitrogen-free extract was calculated (Table 2). According to the Ministerial decree of the Ministry of Agriculture and Land Reclamation (1996), the experimental diets were freely given to the rabbits while they were housed in galvanized wire batteries in a well-ventilated building (naturally through the window). Every cage has an automatic drinker with nipples that provide fresh water at all times. Each rabbit was weighed independently at the beginning of the experiment and then once a week thereafter until they reached marketing age (15 weeks). Rabbits were fed diets as groups, while the feed consumption was daily recorded and kept throughout the trial. All of the rabbits were managed, housed in the same sanitary environment, and given common illness vaccinations.

**Table 1:** Ingredients of the experimental diets (as fed)

Ingredients, %	ML0	ML9	ML12
Mulberry leaves	0.0	2.7	3.6
Clover hay	30.0	27.3	26.4
Barley grain	18.0	18.0	18.0
Soybean meal (44%CP)	14.65	14.65	14.65
Wheat bran	31.25	31.25	31.25
Molasses	3.0	3.0	3.0
DL-Methionine	0.2	0.2	0.2
Vitamins and minerals mixture^*^	0.3	0.3	0.3
Salt	0.2	0.2	0.2
Limestone	1.35	1.35	1.35
Di calcium phosphate	0.45	0.45	0.45
Lysine	0.6	0.6	0.6

*Vitamin A (12000 IU), Vitamin D3 (2200 IU), Vitamin E (10 mg), Vitamin K3 (2.0 mg), Vitamin B1 (1.0 mg), Vitamin B2 (4.0 mg), 1.5 mg Vitamin B6, 0.0010 mg Vitamin B12, 6.7 mg Vitamin Pantothenic acid, 6.67 mg Vitamin B5, 1.07 mg Biotin, 1.67 mg Folic acid, 400 mg Choline chloride, 22.3 mg Zinc, 10 mg Mn, 25 mg Fe, 1.67 mg Cu, 0.25 mg I, 0.033 mg Se, and 133.4 mg Mg are supplied per kilogram of diet. Abbreviation: ML0, basal diet without mulberry leaves (ML); ML9, basal diet with 9% ML; ML12, basal diet with 12% ML substituted from clover hay

**Table 2:** Chemical composition of mulberry leaves (ML), clover hay and experimental diets (% as DM basis)

Chemical composition, %	DM	OM	CP	CF	EE	NFE	Ash
Mulberry leaves	89.76	78.48	16.98	16.39	3.44	41.67	21.52
Clover hay	89.20	85.00	13.43	29.21	2.00	40.36	15.00
ML0	89.60	89.51	15.77	15.57	1.48	56.69	10.49
ML9	88.31	89.31	15.87	14.78	2.18	56.48	10.69
ML12	87.88	89.24	15.90	14.52	2.41	56.41	10.76

Abbreviation: DM, dry matter; OM, organic matter; CP, crude protein; CF, crude fiber; EE, ether extract; NFE, nitrogen free extract; ML0, basal diet without mulberry leaves (ML); ML9, basal diet with 9% ML; ML12, basal diet with 12% ML substituted from clover hay

### Growth performance traits

Live body weight and body weight change of rabbits were weekly recorded, as well as calculations of feed conversion (FC, g feed/g gain) and economic efficacy, were made. According to North (1981), the performance index was calculated as follows: Final body weight, kg/feed conversion ratio × 100. A digestibility trial was performed to ascertain the nutrients’ digestibility coefficient after the approximately nine-week-long experimental period (Fekete 1985).

### Digestibility trial

At the end of feeding trials (15 weeks of age), three digestibility trials were conducted to evaluate the feeding values and nutritional digestibility of the experimental diets. Nine rabbits in total (3 per group) were chosen randomly and housed separately in metabolic cages. Daily feed intake was monitored, and 24 h after the daily feed was given, a quantitative collection of faces was begun. Every day in the morning for 5 days, the faeces of each rabbit were collected, sprayed with 2% boric acid to capture any ammonia emitted, then dried at 60 °C until they reached a constant weight, crushed finely, and stored for chemical analysis. According to (AOAC, 2000**)**. Nutrient digestion coefficients and nutritive values as total digestible nutrients (TDN) and digestible crude protein were estimated according to Fekete (1985). Furthermore, mulberry leaves’ digestible energy (Kcal/kg) was computed using the formula TDN% x 44.3, as stated by Flatt and Schneider (1975).

### Carcass traits

Three rabbits from each group were selected randomly to represent each feeding trial to assess the carcass characteristics. After fasting for around 12 h, each rabbit was weighed to determine its pre-slaughter weight. The rabbits slaughtered were handled humanely. Carcass weight with the head, liver, kidneys, and heart was weighed individually after the entire bleeding and skinning process, and the dressing % was calculated as: Carcass weight / Pre-slaughter weight × 100 according to Blasco and Ouhayoun (1996).

### Blood parameters

A few drops of heparin solution were added to individual blood samples that were obtained from the same slaughtered rabbits and placed into dry, clean centrifuge tubes. To separate the blood plasma, the tubes were centrifuged at 3000 rpm for 20 min. The blood plasma was then stored in a deep freezer at a temperature of roughly − 20 °C until used to determine the blood parameters. Using chemical commercial kits (Bio-Diagnostic, Egypt), the following parameters were calorimetrically determined following the same steps as methods described by manufacturers: Plasma total proteins and creatinine according to Henry (1974), albumin according to Doumas et al. (1971), aspartate aminotransferase and alanine aminotransferase according to Reitman and Frankel (1957), cholesterol according to Thomas (1992), fasting glucose according to Trinder (1969), triglycerides according to Fossati and Prencipe (1982), total lipids according to Zollner and Kirsch (1962) and total antioxidant capacity according to Fang et al. (2011). Globulin was calculated as the difference between total proteins and albumin.

### Economic efficiency

$$\:\mathrm{E}\mathrm{c}\mathrm{o}\mathrm{n}\mathrm{o}\mathrm{m}\mathrm{i}\mathrm{c}\:\mathrm{e}\mathrm{f}\mathrm{f}\mathrm{i}\mathrm{c}\mathrm{i}\mathrm{e}\mathrm{n}\mathrm{c}\mathrm{y},\:\mathrm{\%}\:=\:(\mathrm{N}\mathrm{e}\mathrm{t}\:\mathrm{i}\mathrm{n}\mathrm{c}\mathrm{o}\mathrm{m}\mathrm{e}\:/\:\mathrm{T}\mathrm{o}\mathrm{t}\mathrm{a}\mathrm{l}\:\mathrm{f}\mathrm{e}\mathrm{e}\mathrm{d}\:\mathrm{c}\mathrm{o}\mathrm{s}\mathrm{t})\:\times\:\:100$$

Where: Net income for each rabbit = Price of average weight gain − Total feed cost, average weight gain price = Average weight gain (kg/ rabbit) ×price of 1 kg live body weight, Total feed cost = Average feed intake (kg /rabbit) × price of 1 kg of feed. Relative economic efficiency = Economic efficiency of the treatment ×100 / Economic efficiency of the control group. The cost of each kg of feed was calculated according to the local market price of ingredients at the same time of the experiment (year, 2023). The price of mulberry leaves is the salary of workers who collect the leaves from the land.

### Statistical analysis

Data were statistically analyzed by using SPSS software (2011), one-way analysis of variance, as follows: Y_ij_ = µ + T_ij_+ e_ij_ was the model employed. Where Y_ij_ represents the observation, µ represents the overall mean, Ti represents the impact of treatment, and e_ij_ represents the random error of the experiment. Differences between the means of the experimental groups were found using Duncan’s Multiple Range Test (Duncan 1955). All statements of statistical significance were based on probability (*P* < 0.05).

## Results and discussion

### Chemical composition of feedstuffs and diets

Data from chemical analysis (Table 2) revealed that ML had high levels of crude protein (CP), ether extract (EE) and ash compared to clover hay. Chemical analysis of experimental diets revealed minor variations in the percentages of CP across groups (ML0 up to ML12). The control group (*i.e.* ML0) had the highest value of crude fiber (CF), which decreased steadily from ML0 to ML12%. The current results were comparable to those published by Ustundag and Ozdogan (2023), who reported that the chemical composition of mulberry leaves consisted of 89% DM, 20% CP, 5.5% EE, 11% CF and 11.8% ash. However, these were consistent with those reported by Zeng et al. (2019), who found that the chemical analysis of ML was 17.71% CP, 11.44% CF, and 14.91% ash. The variety, age of the leaves and growing conditions all affect how differently ML is chemically composed. As a result, it is indicated that rabbits could benefit from using dried mulberry leaves as a better source of protein without compromising the hygienic conditions. According to Heuzé et al. (2016), clover hay and ML have similar chemical composition in terms of CP, but ML has higher levels of EE and ash, and this is opposite with found in the current study for CP, which was higher in mulberry leaves compared to clover hay, being 16.98 vs. 13.43%, respectively. Increasing CP and EE and decreasing CF percentage in the diets of ML9 and ML12 compared to the control diet are related to the high content of CP and EE and low CF content in ML compared to clover hay.

### Digestibility and nutritive value

The results obtained in Table 3 showed that the substitution of clover hay with mulberry leaves by 9 and 12% appeared to decrease (*P* < 0.05) dry and organic matter digestibility, while EE increased compared to ML0. There were no significant differences among groups in CP, CF, and nitrogen free extract digestibility. Nutritive values such as total digestible nutrients was higher (*P* < 0.05) in ML0 than ML9, but didn’t differ significantly between ML9 and ML12 or ML0 and ML12. Moreover, digestible crude protein and digestible energy (kcal/kg diets) did not significantly differ across the experimental groups. Increasing EE digestibility with ML9 and ML12 was agreed with Wu et al. (2019) when the mulberry leaf was fed to rabbits instead of alfalfa hay. This outcome could be explained by the slightly greater dietary EE content in the mulberry leaf-based diets as compared to the control diet (Xiccato 2010). Furthermore, Prasad et al. (2003) found that adding 15% of mulberry leaves to the diet in place of all the Lucerne hay did not alter the digestibility of CP or nitrogen free extract, resulting in a similar digestible crude protein and digestible energy, and these were similar to the current study results.

**Table 3:** Nutrients digestibility and nutritive values of experimental diets

Digestibility, %	ML0	ML9	ML12	SEM	*P*-value
Dry matter	72.6^a^	68.7^b^	68.7^b^	0.81	0.044
Organic matter	73.7^a^	69.7^b^	69.6^b^	0.86	0.061
Crude protein	73.4	71.7	71.2	0.74	0.470
Crude fiber	54.9	53.4	52.2	0.75	0.389
Ether extract	55.8^b^	66.2^a^	66.6^a^	1.93	0.004
Nitrogen free extract	79.3	76.6	77.7	0.62	0.201
**Nutritive value**,** %**					
Total digestible nutrients	66.9^a^	65.7^b^	66.4^ab^	0.23	0.097
Digestible crude protein	11.6	11.4	11.3	0.11	0.643
Digestible energy, kcal/kg feed	2965.4	2912.3	2939.3	10.44	0.097

Significant differences (*P* < 0.05) are seen between the means of ^a, b,^ and ^c^ for each parameter in the same row, provided that the superscripts differ. Abbreviations: SEM, standard error of the mean; ML0, basal diet without mulberry leaves (ML); ML9, basal diet with 9% ML; ML12, basal diet with 12% ML in replacement of clover hay

### Growth performance

There were no significant differences in the productive performance of Flanders rabbits (Table 4) among all treatments (ML0, ML9, and ML12). According to Islam et al. (2014), adding 4.5% mulberry to the diet insignificantly increased body weight. Additionally, Shehata et al. (2021) discovered that adding mulberry (Morus Alba) leaf powder to the meal at levels 4 and 8% didn’t affect the growth performance parameters of quail chicks. Similarly, decreasing feed intake with ML12 agreed with another study when rabbits were fed ML as a complete substitute for Lucerne hay (Martínez et al. 2005) and this may affect the digestibility of nutrients (Villamide et al. 1998), but they were similar for CP, CF, and nitrogen free extract digestibility. As the results of feed conversion, revealed that the nutritional content of mulberry leaves is equivalent to that of clover hay and that the dietary habits of the rabbits fed experimental diets were unaffected by the composition of the nutrients, and these findings concur with those of Hou et al. (2020), who discovered that rabbits fed 5, 10, and 15% ML as a replacement for total mixed ration did not exhibit substantially different in average daily gain and feed conversion ratio.

**Table 4:** The effect of mulberry leaves (ML) added to diets on productive performance of growing Flanders rabbits

Item	ML0	ML9	ML12	SEM	*P*-value
Initial BW, g	617	653	607	14.31	0.399
Final BW, g	1845	1906	1839	29.1	0.597
Total BW gain, g	1228	1253	1232	26.3	0.922
Average daily gain, g/rabbit	19.5	19.9	19.6	0.42	0.922
Total feed intake, g/rabbit	5312	5649	5240	-	
daily feed intake, g/rabbit	84.3	89.7	83.2	-	
Feed conversion, feed/gain	4.33	4.51	4.25	0.19	0.894
Performance index, %	42.61	42.26	43.27	1.58	0.818

Abbreviation: SEM, standard error of mean; BW, body weight; ML0, basal diet without mulberry leaves (ML); ML9, basal diet with 9% ML; ML12, basal diet with 12% ML substituted from clover hay

### Carcass traits

The results revealed no significance in carcass traits among all treatments (Table 5) except for kidney and heart percentages, which were significantly higher (*P* < 0.05) in ML0 and ML9 than in ML12 treatments. These findings were in line with Chen and Li (2018), who discovered that replacing mulberry leaves with up to 20% of the total mixed ration of the rabbits’ diets did not affect dressing percentage and with the results of Khan et al. (2020) who reported that ML as a substitute of concentrate feed mixture didn’t influence the relative weight or percentage of liver, heart, and kidney of rabbits.

**Table 5:** The effect of adding mulberry leaves (ML) to diets on carcass traits of growing Flanders rabbits

Items	ML0	ML9	ML12	SEM	*P*-value
Pre-slaughter weight, g	1733	1780	1767	36.4	0.895
Carcass weight, g	999	1105	1051	23.9	0.207
Dressing percentage, %	57.7	62.1	59.5	1.19	0.311
**Edible giblets as a percentage of carcass weight**,** %**					
Liver	2.90	3.06	3.49	0.12	0.112
Heart	0.42^a^	0.46^a^	0.35^b^	0.02	0.011
Kidney	0.73^a^	0.76^a^	0.62^b^	0.03	0.025
Spleen	0.11	0.09	0.10	0.01	0.346
Total edible giblets	4.17	4.37	4.56	0.10	0.341

^a and b^ mean in the same row for each parameter with different superscripts are significantly different (*p* < 0.05). Abbreviation: ML0, basal diet without mulberry leaves (ML); ML9, basal diet with 9% ML; ML12, basal diet with 12% ML substituted from clover hay

### Blood parameters

Table 6 showed no significant differences among different albumin, creatinine, and total protein treatments. The albumin/globulin ratio and cholesterol were less (*P* < 0.05) in ML12 than in ML0 and ML9 treatments. Blood glucose and alanine transaminase concentrations were decreased (*P* < 0.05) with increasing mulberry leaf percentages in the Flanders rabbits’ diet. The aspartate transaminase concentration was lower (*P* < 0.05) in ML0 compared to other treatments. Total lipid concentration recorded lower (*P* < 0.05) levels in ML0 and ML12 than in ML9. The ML12 revealed a high (*P* < 0.05) total antioxidant capacity as compared to ML9. All blood profiles were within the normal range for healthy rabbits (Abdulkareem et al. 2020) and were consistent with the findings of Chen and Li (2018) for rabbits given varying amounts of mulberry leaves as a substitute for whole meals, and these findings demonstrate the beneficial effects of mulberry leaves on rabbit health improvements. According to Jeszka-Skowron et al. (2014) and Pham et al. (2017), mulberry leaves contain phytochemicals that are potent antioxidants that induce antioxidant enzymes in rabbits; these phytochemicals include the alkaloid 1-deoxynojirimycin and flavonoids.

**Table 6:** Blood parameters of the Flanders rabbits as affected by adding mulberry leaves (ML)

	ML0	ML9	ML12	SEM	*P*-value
Total protein, g/dl	6.41	5.97	6.82	0.16	0.087
Albumin, g/dl	3.55	3.46	3.27	0.11	0.642
Globulin, g/dl	2.86^b^	2.51^c^	3.55^a^	0.15	0.000
Albumin/Globulin ratio	1.24^a^	1.38^a^	0.92^b^	0.08	0.018
Creatinine mg/dl	0.99	0.99	1.02	0.02	0.760
Glucose, mg/dl	80.40^a^	74.21^b^	70.66^c^	1.45	0.000
AST, U/L	55.80^b^	66.60^a^	62.40^a^	1.72	0.004
ALT, U/L	86.63^a^	79.35^b^	72.87^c^	2.17	0.004
Cholesterol, mg/dl	161.91^a^	159.50^a^	136.19^b^	4.16	0.000
Triglyceride, mg/dl	78.31^a^	64.58^b^	63.78^b^	2.48	0.001
Total lipid, mg/dl	445.45^b^	618.18^a^	413.64^b^	32.50	0.000
TAC, mmol/l	0.99^ab^	0.96^b^	1.01^a^	0.01	0.023

^a, b and c^ mean in the same column with different superscripts are significantly (*P* < 0.05) different. Abbreviation: ALT, alanine transaminase; AST, aspartate transaminase; TAC, total antioxidant capacity; SEM, standard error of mean; ML0, basal diet without mulberry leaves (ML); ML9, basal diet with 9% ML; ML12, basal diet with 12% ML substituted from clover hay

### Economic efficiency

The results in Table 7 showed that adding 12% mulberry leaves to growing rabbit diets increased net revenue. The rabbits fed a diet containing 12% mulberry leaves had the lowest total feed cost per rabbit, followed by those fed a control diet and then those fed ML9 (56.73 LE). Meanwhile, the group fed ML12 recorded the best net income, economic efficiency, and relative economic efficiency compared to ML9 and ML0. The length of the growing season, final body weight, and feeding expenses are typically the most crucial elements in achieving the highest possible levels of meat production efficiency. The results reported that groups fed ML12 achieved better performance compared to other groups. In general, the most crucial elements in achieving the highest efficiency values of meat production with this group are the ultimate body weight and the cost of feeding. Because clover hay is quite expensive and mulberry leaves are inexpensive, the price of kg feed generated by the experimental 12% group resulted in a profit. In light of our findings, additional research is required to elucidate the advantages of mulberry leaves in the diets of rabbits with varying degrees of substitution. Mohamed (1999) discovered that substituting peanut hay, as a cheap ingredient, for clover hay reduced the expense of feeding and increased economic efficiency.

**Table 7:** Economic efficiency of experimental diets

Items	ML0	ML9	ML12
Final body weight, kg	1.85	1.91	1.84
Total weight gain/rabbit, kg	1.23	1.25	1.23
Price/ kg live weight, LE	80.00	80.00	80.00
Price of total weight gain, LE	98.4	100.0	98.4
Total feed intake/rabbit, kg	5.31	5.65	5.24
*Price of kg feed, LE	10.22	10.04	9.98
Total feed cost/rabbit, LE	54.27	56.73	52.30
Net income/kg meat produced	44.13	43.27	46.10
Economic efficiency	0.81	0.76	0.88
Relative economic efficiency (%)	100.0	93.83	108.64

Based on the local market price of ingredients at the time of the experiment (the year2023), Mulberry leaves = 200 LE. /Ton (the salary of labors who collected the leaves), clover hay = 7000 LE. /Ton and concentrate feed mixture (CFM) = 11,600 LE/ton. Abbreviation: LE, Egyptian pound; ML0, basal diet without mulberry leaves (ML); ML9, basal diet with 9% ML; ML12, basal diet with 12% ML substituted from clover hay

## Conclusions

Mulberry leaves (ML) can safely alternatively replace clover hay in growing rabbit diets up to 12%, improving growth and reducing feed costs. Further research should explore higher inclusion levels and their impact on reproduction, meat quality, and sustainability.

## Data Availability

The data that support the findings of this study are available from the corresponding author upon reasonable request.
